# Additions to Italian Pleosporinae, including *Italica heraclei* sp. nov.

**DOI:** 10.3897/BDJ.9.e59648

**Published:** 2021-01-18

**Authors:** Subodini N. Wijesinghe, Yong Wang, Laura Zucconi, Monika C. Dayarathne, Saranyaphat Boonmee, Erio Camporesi, Dhanushka N. Wanasinghe, Kevin D. Hyde

**Affiliations:** 1 Department of Plant Pathology, Agriculture College, Guizhou University, Guiyang, Guizhou Province, 550025, China Department of Plant Pathology, Agriculture College, Guizhou University Guiyang, Guizhou Province, 550025 China; 2 Center of Excellence in Fungal Research, Mae Fah Luang University, Chiang Rai 57100, Thailand Center of Excellence in Fungal Research, Mae Fah Luang University Chiang Rai 57100 Thailand; 3 School of Science, Mae Fah Luang University, Chiang Rai 57100, Thailand School of Science, Mae Fah Luang University Chiang Rai 57100 Thailand; 4 Department of Ecological and Biological Sciences, University of Tuscia, Largo dell'Università snc, 01100, Viterbo, Italy Department of Ecological and Biological Sciences, University of Tuscia, Largo dell'Università snc, 01100 Viterbo Italy; 5 A.M.B. Gruppo Micologico Forlivese “Antonio Cicognani”, Via Roma 18, Forlì, Italy A.M.B. Gruppo Micologico Forlivese “Antonio Cicognani”, Via Roma 18 Forlì Italy; 6 CAS Key Laboratory for Plant Biodiversity and Biogeography of East Asia (KLPB), Kunming Institute of Botany, Chinese Academy of Sciences, Kunming 650201, Yunnan, China CAS Key Laboratory for Plant Biodiversity and Biogeography of East Asia (KLPB), Kunming Institute of Botany, Chinese Academy of Sciences Kunming 650201, Yunnan China; 7 East and Central Asia Regional Office, World Agroforestry Centre (ICRAF), Kunming 650201, Yunnan, China East and Central Asia Regional Office, World Agroforestry Centre (ICRAF) Kunming 650201, Yunnan China; 8 Honghe Center for Mountain Futures, Kunming Institute of Botany, Chinese Academy of Sciences, Honghe County, Yunnan, China Honghe Center for Mountain Futures, Kunming Institute of Botany, Chinese Academy of Sciences Honghe County, Yunnan China; 9 Innovative Institute of Plant Health, Zhongkai University of Agriculture and Enginnering, Haizhu District, Guangzhou 510225, China Innovative Institute of Plant Health, Zhongkai University of Agriculture and Enginnering Haizhu District, Guangzhou 510225 China

**Keywords:** one new species, Ascomycota, Dothideomycetes, integrative taxonomy, morphology, phylogeny

## Abstract

**Background:**

In the last few years, many microfungi—including plant-associated species—have been reported from various habitats and substrates in Italy. In this study of pleosporalean fungi, we researched terrestrial habitats in the Provinces of Arezzo (Tuscany region), Forlì-Cesena and Ravenna (Emilia-Romagna region) in Italy.

**New information:**

Our research on Italian pleosporalean fungi resulted in the discovery of a new species, *Italica heraclei* (Phaeosphaeriaceae). In addition, we present a new host record for *Pseudoophiobolus mathieui* (Phaeosphaeriaceae) and the second Italian record of *Phomatodes nebulosa* (Didymellaceae). Species boundaries were defined, based on morphological study and multi-locus phylogenetic reconstructions using Maximum Likelihood and Bayesian Inference analyses. Our findings expand the knowledge on host and distribution ranges of pleosporalean fungi in Italy.

## Introduction

A number of prominent scholars contributing to the foundation of fungal classification were of Italian origin. Among the most important mycologists of the 19^th^ century are Giuseppe De Notaris and Pier Andrea Saccardo, who were the earliest mycologists to validate microscopic characteristics as important features in fungal taxonomy (Onofri et al. 1999). Currently, fungal taxonomy benefits from a combination of morphology and DNA-based molecular analyses to resolve species limits (e.g. Alors et al. 2016, Skrede et al. 2017, Haelewaters and De Kesel 2020). In the last few years, a high number of microfungal taxa have been recorded in different Italian habitats (Jensen et al. 2010, Rodolfi et al. 2016,Thambugala et al. 2017,Wanasinghe et al. 2018a, Liu et al. 2019, Marin-Felix et al. 2019, Hyde et al. 2020b). Currently, a database (https://italianmicrofungi.org/) for plant-associated Italian microfungi is being developed with past, present and upcoming studies being added.

The order Pleosporales is amongst the most family-rich ones in Dothideomycetes (Mugambi and Huhndorf 2009, Taylor et al. 2015, Brahmanage et al. 2020), with 91 families and 566 genera (Hongsanan et al. 2020, Wijayawardene et al. 2020). Luttrell (1955) suggested that Pleosporales should contain dothideomycetous species with perithecioid ascomata and pseudoparaphyses amongst the asci. After investigations by Luttrell (1973) and Barr (1983), the Pleosporales order was established by Barr (1987), based on Pleosporaceae with the type species *Pleospora herbarum*. The order includes taxa characterised by perithecioid ascomata with perithecia that have a papillate apex and ostiole, with or without periphyses, cellular pseudoparaphyses, fissitunicate asci and ascospores with variable pigmentation, septation and shape and usually with bipolar asymmetry (Barr 1987, Hyde et al. 2013). In this study, we investigated three fungal taxa in pleosporalean families, two taxa belonging to Phaeosphaeriaceae and one to Didymellaceae.

Phaeosphaeriaceae was introduced by Barr (1979). The family was typified by *Phaeosphaeria* and *P. oryzae* is the type species (Hongsanan et al. 2020). Members of this family can be saprotrophic, endophytic, pathogenic on economically-important plants and crops and hyper-parasitic on living plants and humans (Kirk et al. 2010, Bakhshi et al. 2019, Phookamsak et al. 2014, Senanayake et al. 2018, Roels et al. 2020). Phaeosphaeriaceous species associated with monocotyledons have been often described as having small to medium-sized ascomata and septate, ellipsoidal to fusiform or filiform ascospores (Zhang et al. 2012, Hyde et al. 2013, Hyde et al. 2016). Some species of Phaeosphaeriaceae have been recorded from dicotyledonous plants (Ariyawansa et al. 2015a, Hyde et al. 2016, Hyde et al. 2020b, Brahmanage et al. 2020).

Didymellaceae is another family in Pleosporales introduced by De Gruyter et al. (2009) to accommodate *Ascochyta*, *Didymella* (type), *Phoma* and *Phoma*-like species (Chen et al. 2015, Chen et al. 2017, Hongsanan et al. 2020). It is a species-rich family containing numerous plant pathogenic, saprotrophic and endophytic species associated with a wide range of hosts (Aveskamp et al. 2008, Aveskamp et al. 2010, Wanasinghe et al. 2018a, Hou et al. 2020). Species of Didymellaceae are cosmopolitan and have been reported from inorganic materials, water, air, soil and different environments, such as deep-sea sediments, deserts and karst caves (Wanasinghe et al. 2018a, Hongsanan et al. 2020, Hou et al. 2020).

Currently, a total of 83 and 35 genera are accounted for Phaeosphaeriaceae and Didymellaceae, respectively (Hongsanan et al. 2020, Wijayawardene et al. 2020). New additions of phaeosphaeriaceous and didymellaceous species have been recorded from Italian localities in the last few years, from multiple hosts, substrates and geographical locations (Ariyawansa et al. 2015b, Marin-Felix et al. 2019, Farr and Rossman 2020). Here, we present the characterisation of three fungal strains isolated from dead attached stems of different dicotyledon hosts collected in Italy.

## Materials and methods

**Sample collection, morphological studies and specimen deposition**

Strains were isolated from dead stems of different host plants belonging to Apiaceae, Asteraceae and Urticaceae (dicotyledons) collected in the Provinces of Arezzo, Forlì-Cesena and Ravenna (Italy) from September to December 2018. Samples were preserved in sterile Ziploc bags in the laboratory at 18°C. Macromorphological characters of the samples were observed using a Motic SMZ 168 compound stereomicroscope and micromorphology was examined from hand-sectioned structures using a Nikon ECLIPSE 80i compound stereomicroscope, equipped with a Canon 600D digital camera. The measurements of photomicrographs were obtained using Tarosoft (R) Image Frame Work version 0.9.7. Images were edited with Adobe Photoshop CS6 Extended version 13.0.1 software (Adobe Systems, San Jose, California).

Single-spore isolation was carried out as described by Chomnunti et al. (2014). Germinated spores were aseptically transferred into fresh potato dextrose agar medium (PDA, Merck KGaA of Darmstadt, Germany). Culture plates were incubated at 18°C for six weeks and inspected every week. Herbarium specimens are preserved at Mae Fah Luang University Herbarium (MFLU) in Chiang Rai, Thailand. All living cultures are deposited at Mae Fah Luang Culture Collection (MFLUCC). Facesoffungi and Index Fungorum numbers for new taxa were obtained (Jayasiri et al. 2015, Index Fungorum 2020).

The administrative boundaries of Italy and geocodes for collecting sites related to our newly-isolated species were mapped by using QGIS version 3.14 (QGIS Geographic Information System, Open Source Geospatial Foundation Project. http://qgis.org/). Geocodes of collecting locations were confirmed with GoogleEarthPro version 7.3.3 (the data providers were: Image Landsat/Copernicus, Data SIO, NOAA, US. Navy, NGA, GEBCO, US Dept. of State Geographer, https://www.google.com/earth/). The data files (.cvs and .shp) for administrative boundaries were downloaded from DIVA-GIS for mapping and geographic data analysis (Hijmans et al. 2001, https://www.diva-gis.org/).

### DNA extraction, PCR amplification, sequencing and molecular analyses

The methodology for DNA extraction, PCR, gel electrophoresis and sequencing was followed, as detailed in Dissanayake et al. (2020). The genomic DNA was extracted from fresh mycelium using EZgeneTM Fungal gDNA extraction Kit GD2416 (Biomiga, Shanghai, China), following the guidelines of the manufacturer. DNA sequences were obtained for the internal transcribed spacer region (ITS1, 5.8S, ITS2), the small subunit (SSU) and large subunit (LSU) of the nuclear ribosomal RNA gene, translation elongation factor 1-a (TEF) and ß-Tubulin (TUB2). PCR thermal cycle programmes for each locus region are presented in Table 1. Purification and sequencing were outsourced to the SinoGenoMax Sanger sequencing laboratory (Beijing, China). Newly-generated sequences were submitted to NCBI GenBank (https://www.ncbi.nlm.nih.gov/genbank/).

Contig sequences were checked with BLAST searches in NCBI for primary identifications. Sequences for phylogenetic analyses were downloaded from GenBank following Hyde et al. (2020b). Single and multiple alignments were generated with MAFFT version 7 (Katoh and Standley 2013, Katoh et al. 2019). When manual improvement was needed, BioEdit version 7.0.5.2 software was used (Hall 1999). Two separate phylogenetic analyses were conducted: Maximum Likelihood (ML) and Bayesian Inference (BI). The following concatenated datasets were analysed: for Didymellaceae: ITS, LSU, RPB2, TUB2; for Phaeosphaeriaceae: SSU, ITS, LSU, TEF (*sensu* Hyde et al. 2020b).

Phylogenetic analyses were run on the CIPRES Science Gateway portal (Miller et al. 2012). ML trees were generated for the final concatenated alignment by using RAxML-HPC2 on XSEDE (v. 8.2.10) tool (Stamatakis 2014) under the GTR+GAMMA substitution model. Bootstrapping was done with 1,000 replicates. For BI, MrModeltest version 2.3 (Nylander 2004) was run under the Akaike Information Criterion implemented in PAUP version 4.0b10 (Swofford 2003) to estimate the best evolutionary model, resulting in GTR+I+G as the best-fit model for each locus. The BI analysis was computed with MrBayes version 3.2.6 (Ronquist et al. 2012). Six simultaneous Markov chains were run for 3,000,000 generations (Didymellaceae) or 2,000,000 generations (Phaeosphaeriaceae). Trees were sampled every 1000 generations, ending the run automatically when the standard deviation of split frequencies dropped below 0.01. Phylogenetic trees were visualised with FigTree version 1.4.0 (Rambaut 2012) and edited in Microsoft PowerPoint (2016). The final alignments and trees were deposited in TreeBASE, with submission ID 27224 for Didymellaceae (http://purl.org/phylo/treebase/phylows/study/TB2:S27224) and submission ID 27225 for Phaeosphaeriaceae (http://purl.org/phylo/treebase/phylows/study/TB2:S27225).

### Phylogenetic analyses

**Figure 1.**
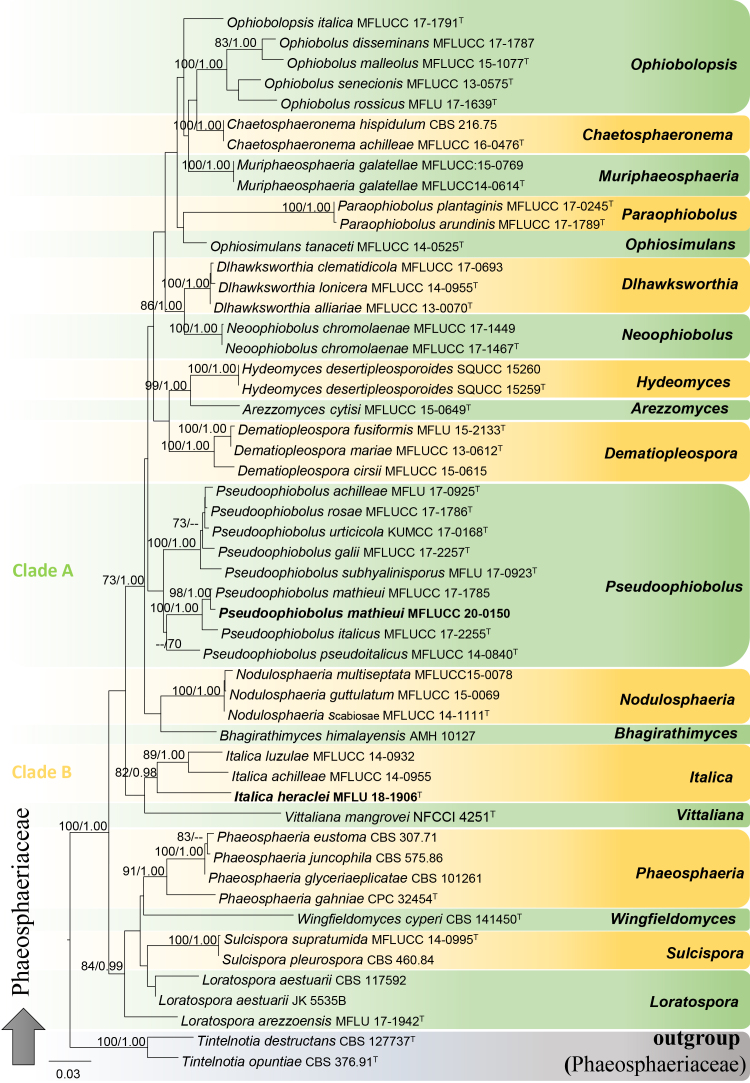
Phylogeny of the family Phaeosphaeriaceae, reconstructed from the combined SSU–ITS–LSU–TEF dataset. *Tintelnotia destructants* (CBS 127737) and *T. opuntiae* (CBS 376.91) serve as outgroup taxa. ML = 70 and PP = 0.95 are presented above each node. The new isolates are indicated in bold; ^T^ = type strains. The scale bar represents the expected number of nucleotide substitutions per site.

For the phylogenetic analysis of Phaeosphaeriaceae, *Tintelnotia destructants* (CBS 127737) and *T. opuntiae* (CBS 376.91) were selected as outgroup taxa. The dataset comprised 52 taxa, including our new isolates. The final concatenated dataset comprised 3307 characters including gaps. ML and BI analyses resulted in similar tree topologies. The final RAxML tree is shown in Fig. 1 (-lnL = 13938.841645). For the BI analysis, 20% of generations were discarded, resulting in 1583 remaining trees, from which 50% consensus trees and Posterior Probabilities (PP) were calculated.

In our phylogenetic analyses, the new species *Italica heraclei* (MFLUCC 20-0227) formed a phylogenetically-distinct lineage with high support (82 ML/0.98 PP) (Clade B, Fig. 1), within genus *Italica*. The generic placements of related *Italica* species were similar to the analysis performed by Hyde et al. (2020b). In addition, our Italian isolate of *Pseudoophiobolus mathieui* (MFLUCC 20-0150) and the ex-type strain of *P. mathieui* (MFLUCC 17-1785) clustered together with high support (98 ML/1.00 PP) (Clade A, Fig. 1). *Pseudoophiobolus mathieui* was placed sister to *Pseudoophiobolus italica*, similar to the phylogenetic analysis performed by Phookamsak et al. (2017).

**Figure 2.**
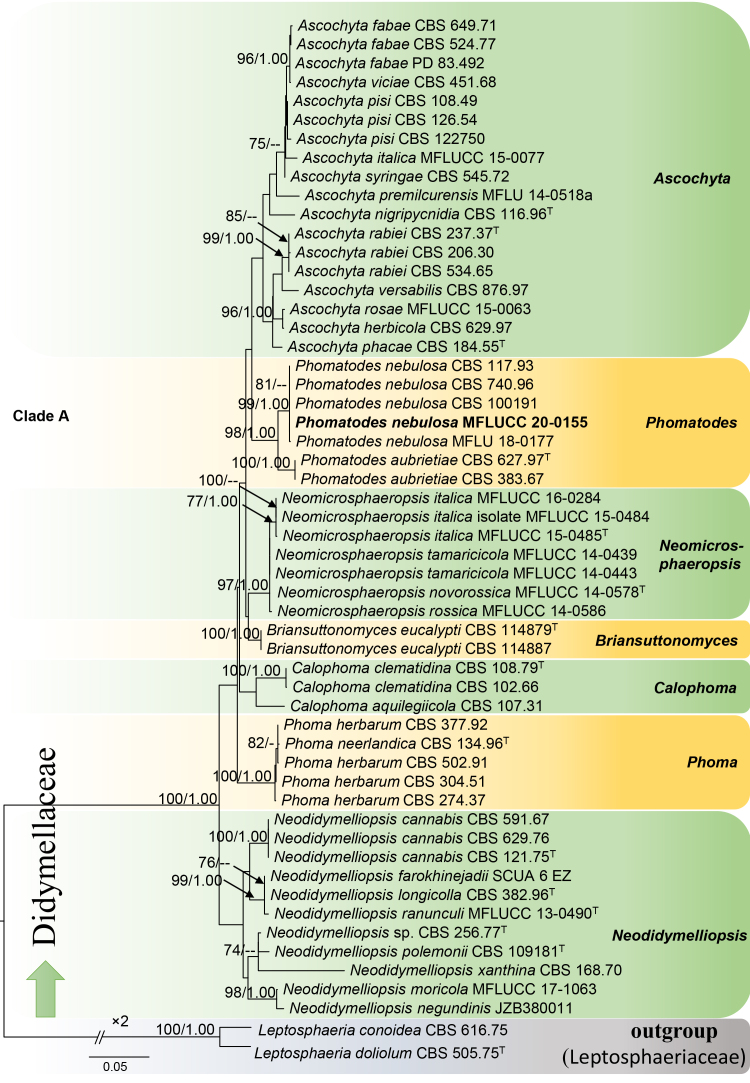
Phylogeny of the family Didymellaceae, reconstructed from the combined ITS–LSU–RPB2–TUB2 dataset. *Leptosphaeria conoidea* (CBS 616.75) and *L. doliolum* (CBS 505.75) serve as outgroup taxa. ML = 70 and PP = 0.95 are presented above each node. The new isolate is indicated in bold; ^T^ = type strains. The scale bar represents the expected number of nucleotide substitutions per site.

For Didymellaceae, *Leptosphaeria conoidea* (CBS 616.75) and *L. doliolum* (CBS 505.75) were selected as outgroup taxa. The concatenated ITS–LSU–RPB2–TUB2 dataset comprised 55 taxa, including our new isolates. The final dataset comprised 2154 characters including gaps. ML and BI analyses resulted in similar tree topologies. The final RAxML tree is shown in Fig. 2 (-lnL = 6261.962009). For the BI analysis, 20% of generations were discarded, resulting in 2401 remaining trees, from which 50% consensus trees and PP were calculated.

In our phylogenetic analyses, the newly-isolated Italian strain MFLUCC 20-0155 was grouped in *Phomatodes* (Clade A, Fig. 2), with high support values (99 ML/1.00 PP) with other strains of *Phomatodes nebulosa*: CBS 117.93, CBS 740.96, CBS 100191 and MFLU 18-0177. This, combined with the morphological study (below), confirmed the identity of our isolate as *Phomatodes nebulosa*.

## Taxon treatments

### Italica heraclei

Wijes., Yong Wang bis, Camporesi & K.D. Hyde sp. nov.

D231C179-A0CB-5A11-8B50-2879901E3BA6

IF 557859

FoF 09223

#### Materials

- **Type status:** Holotype. **Occurrence:** recordedBy: Erio Camporesi; **Taxon:** scientificName: *Italica heraclei* Wijes., Yong Wang bis, Camporesi & K.D. Hyde, sp. nov.; kingdom: Fungi; phylum: Ascomycota; class: Dothideomycetes; order: Pleosporales; family: Phaeosphaeriaceae; genus: Italica; specificEpithet: heraclei; taxonRank: species; **Location:** stateProvince: Province of Forlì-Cesena [FC]; county: Italy; municipality: near Ranchio; **Identification:** identifiedBy: S.N. Wijesinghe; **Event:** year: 2018; month: 09; day: 10; habitat: on a dead aboveground stem of *Heracleum sphondylium* (Apiales, Apiaceae); fieldNotes: Terrestrial; **Record Level:** institutionID: MFLU 18-1906; institutionCode: Mae Fah Luang University Herbarium (MFLU); ownerInstitutionCode: IT 4028
- **Type status:** Other material. **Record Level:** type: ex-type living culture; collectionID: MFLUCC 20-0227; collectionCode: Mae Fah Luang Culture Collection (MFLUCC)

#### Description

**Figure 3.**
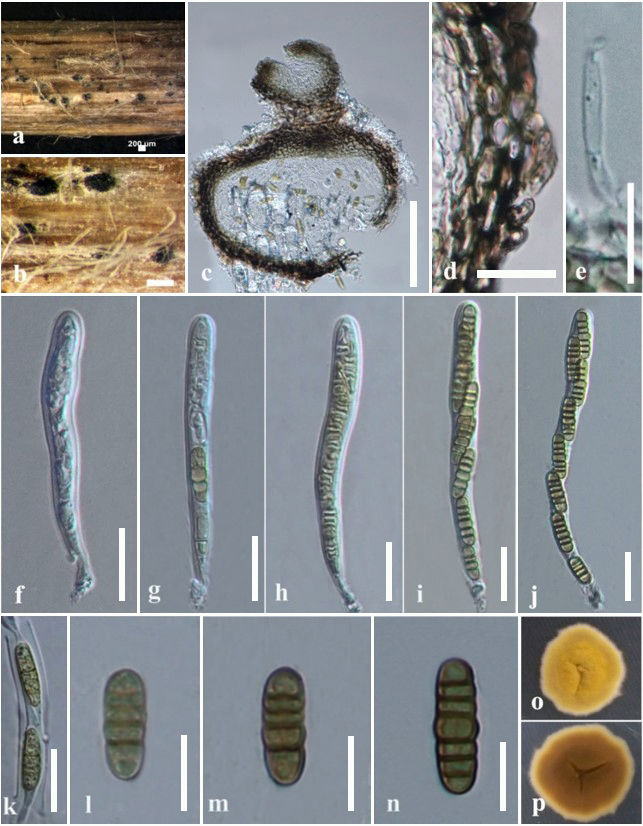
*Italica heraclei* (MFLU 18-1906). **a-b.** Ascomata on a dead stem of *Heracleum sphondylium* (Apiales, Apiaceae). **c.** Section of an ascoma. **d.** Peridium. **e.** Pseudoparaphyses. **f-j.** Asci. **k-n.** Ascospores. **o-p.** Colonies on PDA from upper (**o**) and lower (**p**) sides. Scale bars: a-b = 200 µm, c = 100 µm, d-j = 20 µm, k-n = 10 µm.

Saprobic on dead aboveground stem of *Heracleum sphondylium* L. (Apiales, Apiaceae). **Sexual morph**: *Ascomata* (Fig. 3a-c) 250–280 × 230–250 µm (x¯ = 257 × 237 µm, n = 10), immersed to erumpent, solitary scattered, sessile, globose to subglobose, uniloculate, dark brown to black, coriaceous, ostiolate. *Ostiole* (Fig. 3c) papillate, 70–85 µm long, 80–100 µm wide, central, comprising blackish-brown to pale brown or hyaline cells. *Peridium* (Fig. 3d) 10–25 µm (x¯ = 16 µm, n = 15) wide, thin-walled, composed of 4–6 cell layers, outermost layers heavily pigmented, comprising dark brown to pale brown cells of *textura angularis*. *Hamathecium* comprising numerous, 2–3 µm wide (x¯ = 2.5 µm, n = 10), filamentous, branched pseudoparaphyses (Fig. 3e) with distinct septa. *Asci* (Fig. 3f-k) 80–120 × 8–9 µm (x¯ = 100 × 8.5 µm, n = 10), 8-spored, bitunicate, fissitunicate, cylindrical, apically rounded with thick-walled, minute ocular chamber, short pedicellate. *Ascospores* (Fig. 3l-n) 13–22 × 4–5.5 µm (x¯ = 18 × 5 µm, n = 30), overlapping, uniseriate, ellipsoidal to subcylindrical, 4–6 transversely septate, vertically aseptate, with rounded ends, widest at the middle cell when matured, constricted at the septa, initially hyaline, becoming yellowish-brown at maturity, smooth-walled, mucilaginous sheath absent. **Asexual morph**: Undetermined.

Culture characteristics: Ascospores germinating on MEA (malt extract agar) within 2 days, from single-spore isolation. Colonies (Fig. 3o-p) on PDA reaching 5–10 mm diam. after 28 days at 18°C, circular, entire edge, flat, dense, bright yellow in both upper and lower sides.

GenBank accession numbers (ex-MFLU 18-1906^T^): SSU = MT881671, ITS = MT881676, LSU = MT881653, TEF = MT901290

#### Etymology

Etymology: *heraclei*, referring to the host genus *Heracleum* from which the strains were isolated.

#### Notes

Notes: *Italica heraclei* (holotype MFLU 18-1906) was isolated from a dead aerial stem of *Heracleum sphondylium* (Apiales, Apiaceae), whereas *I. achilleae* (MFLUCC 14-0955) and *I. luzulae* (MFLUCC 14-0932) were previously isolated from *Achillea millefolium* (Asterales, Asteraceae) and *Luzula* sp. (Poales, Juncaceae), respectively. The strains of *Italica heraclei* (MFLUCC 20-0227) and *I. achilleae* (MFLUCC 14-0955) were collected from the same Province, Forlì-Cesena; *Italicaluzulae* (MFLUCC 14-0932) was collected from Trento Province (Ariyawansa et al. 2015b, Wanasinghe et al. 2018b).

*Italica heraclei* (MFLUCC 20-0227) shows morphological characters that are typical for the genus, including coriaceous ascomata; filamentous, branched and septate pseudoparaphyses; and hyaline to yellowish-brown ascospores. *Italica heraclei* differs from other *Italica* species by its cylindrical asci and vertically aseptate (Fig. 3) and uniseriate-arranged ascospores in asci.

From the comparison of the SSU, ITS, LSU and TEF sequences of *I. heraclei* (MFLUCC 20-0227) and *I. luzulae* (MFLUCC 14-0932, type species) strains, we detected 3/949 (0.31%), 67/517 (12.95%), 20/796 (2.51%) and 32/619 (5.16%) differences, respectively. From the comparison of SSU, ITS, LSU and TEF nucleotides of *I. heraclei* and *I. achilleae* (MFLUCC 14-0955), we found 1/950 (0.1%), 64/517 (12.37%), 7/796 (1.13%) and 28/619 (4.52%) differences, respectively. According to the results of our integrative taxonomy approach, we described *I. heraclei* (MFLUCC 20-0227) as a new species.

### Pseudoophiobolus mathieui

(Westend.) Phookamsak., Wanas., S.K. Huang, Camporesi & K.D. Hyde (2017)

29E4C2F0-5ADA-5F93-89E0-A48495626C05

IF554183

FoF 03804

Pseudoophiobolus mathieui *Sphaeria mathieui*
Basionym: *Sphaeria mathieui* Westend., Bull. Acad. R. Sci. Belg., Cl. Sci., sér. 2: no. 5 (1859)

#### Materials

- **Type status:** Other material. **Occurrence:** recordedBy: Erio Camporesi; **Taxon:** kingdom: Fungi; phylum: Ascomycota; class: Dothideomycetes; order: Pleosporales; family: Phaeosphaeriaceae; genus: Pseudoophiobolus; specificEpithet: mathieui; taxonRank: species; **Location:** stateProvince: Province of Ravenna; county: Italy; municipality: near Brisighella; **Identification:** identifiedBy: S.N. Wijesinghe; **Event:** year: 2018; month: 9; day: 10; habitat: on a dead areail stem of *Artemisia* sp. (Asterales, Asteraceae); fieldNotes: Terrestrial; **Record Level:** institutionID: MFLU 18-1907; institutionCode: Mae Fah Luang University Herbarium (MFLU); ownerInstitutionCode: IT4031
- **Type status:** Other material. **Record Level:** type: living culture; collectionID: MFLUCC 20-0150; collectionCode: Mae Fah Luang Culture Collection (MFLUCC)

#### Description

**Figure 4.**
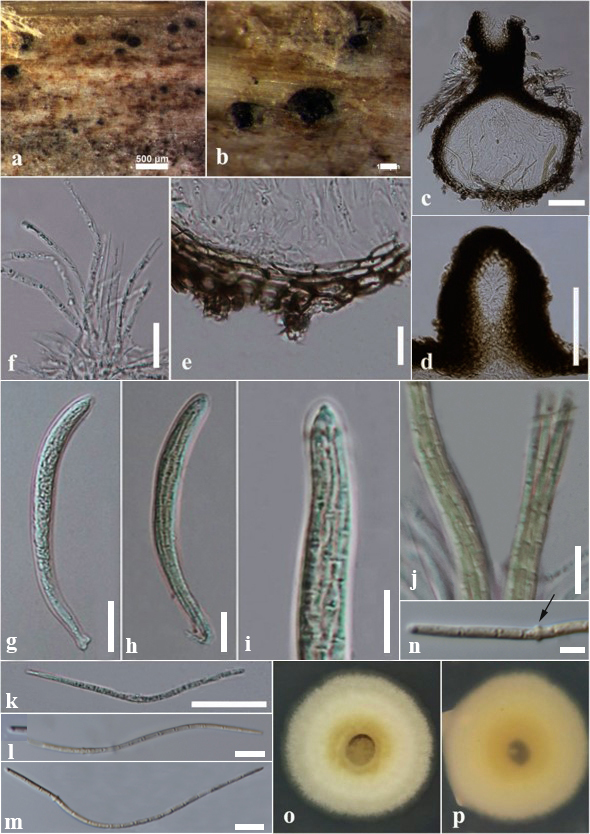
*Pseudoophiobolus mathieui* (MFLU 18-1907). **a-b.** Ascomata on dead host surface of *Artemisia* sp. (Asterales, Asteraceae). **c.** Section of an ascoma. **d.** Close-up of ostiole. **e.** Peridium. **f.** Pseudoparaphyses. **g-j.** Asci. **k-m.** Ascospores. **n.** Ascospore with a swollen point (arrow). **o-p.** Colonies on PDA from upper (**o**) to lower (**p**) sides. Scale bars: b, d = 100 µm, c, f = 50 µm, e, g, h, l, m = 20 µm, i, j = 10 µm, n = 5 µm.

Saprobic on dead aerial stem of *Artemisia* sp. (Asterales, Asteraceae). **Sexual morph**: *Ascomata* (Fig. 4a-b, c - with ostiole) 170–300 × 140–250 µm (x¯ = 200 × 177 µm, n = 10), solitary, scattered, dark brown to black, semi-immersed to erumpent, sessile, globose to subglobose, uni-loculate, coriaceous, ostiolate and papillate. *Papilla* (Fig. 4d) 70–150 × 60–120 µm, mammiform to oblong, with a rounded to truncate apex, thick walled, composed of several layers, brown to dark brown cells of *textura angularis*, ostiole central, single and without periphyses. *Peridium* (Fig. 4e) 15–35 µm (x¯ = 20 µm, n = 15), brown to black, thick-walled, pseudoparenchymatous cells, composed of 4–6 cell layers, outer layers composed of dark brown loosely packed cells of *textura angularis*, inner layers composed of light brown to hyaline flattened cells of textura prismatica. *Hamathecium* comprising numerous, 1.5–2.5 µm wide (x¯ = 2 µm, n = 15), filamentous, distinctly septate, cellular pseudoparaphyses (Fig. 4f) with guttules, slightly constricted at the septa, anastomosing at the apex, embedded in a hyaline gelatinous matrix. *Asci* (Fig. 4g-j) 100–150 × 6–9 µm (x¯ = 132 × 8 µm, n = 15), 8-spored, bitunicate, fissitunicate, cylindrical to cylindrical-clavate, short furcate pedicel, apically rounded, well-developed ocular chamber. *Ascospores* (Fig. 4k-m) 120–150 × 2–3 µm (x¯ = 131 × 2.8 µm, n = 25), fasciculate, lying parallel or spiral at the centre, scolecosporous, filiform or filamentous, narrowly rounded towards the ends, slightly swollen at the middle of 4^th^ or 5^th^ cell from the apex (Fig. 4n), yellowish to yellowish brown, 15–18 septate and not constricted at the septa, smooth-walled. **Asexual morph**: Undetermined.

Culture characteristics: Ascospores germinating on PDA within 4 days, from single-spore isolation. Colonies (Fig. 4o-p) on PDA reaching 10–15 mm diam. after 14 days at 16°C, circular, entire edge, flat, dense, pale yellow in both upper and lower centres, white at the edges in both sides.

GenBank accession numbers (ex-MFLUCC 20-0150): SSU = MT880290, ITS = MT880294, LSU = MT880292, TEF = MT901292

#### Notes

*Pseudoophiobolus* was introduced by Phookamsak et al. (2017) to accommodate *Ophiobolus*-like taxa, including *P. mathieui*, characterised by ascospores that are subhyaline to pale yellowish or yellowish, with a swollen cell, lacking terminal appendages and not separating into part spores. Both the new Italian strain (MFLUCC 20-0150) and the previously-isolated ex-type strain of *P. mathieui* (MFLUCC 17-1785) were collected from the Province of Forlì-Cesena, on *Artemisia* sp. (Asterales, Asteraceae) and *Salvia* sp. (Lamiales, Lamiaceae), respectively. Further records were reported for the same Province on *Origanum vulgare* (Lamiales, Lamiaceae) and *Ononis spinosa* (Fabales, Fabaceae) (Phookamsak et al. 2017). Characteristics of our material resemble the holotype (Phookamsak et al. 2017). The holotype of *P. mathieui* (MFLUCC 17-1785) and our newly-isolated strain (MFLUCC 20-0150) were similar in ascomata, ostiole, peridium and asci, but the ascomatal ostiole of MFLUCC 20-0150 was composed of cells of *textura angularis*, whereas, in MFLUCC 17-1785, the cells were of *textura angularis* to *textura prismatica* (Fig. 4).

From a comparison of ITS and LSU sequences between *P. mathieui* (type) and MFLUCC 20-0150 strain, both were identical. However, seven nucleotide differences (1.13%) were found between the TEF sequences of two strains. Following the integrative taxonomic approach with both morphological data and molecular phylogenetic analyses, we conclude that our new collection is *Pseudoophiobolus mathieui* and represents a new host record on *Artemisia* sp. (Asterales, Asteraceae).

### Phomatodes nebulosa

(Pers.) Qian Chen & L. Cai, Stud. Mycol. 82: 191 (2015)

A80883D5-4F7B-5E04-B9B9-28795D08F153

IF 814134

FoF 06803

Phomatodes nebulosa *Sphaeria nebulosa*
= *Sphaeria nebulosa* Pers., Observ. mycol. (Lipsiae) 2: 69 (1800) [1799]

#### Materials

- **Type status:** Other material. **Occurrence:** recordedBy: Erio Camporesi; **Taxon:** namePublishedIn: *Phomatodes nebulosa* (Pers.) Qian Chen & L. Cai, *Stud. Mycol.* 82: 191 (2015); kingdom: Fungi; phylum: Ascomycota; class: Dothideomycetes; order: Pleosporales; family: Didymellaceae; genus: Phomatodes; specificEpithet: nebulosa; taxonRank: species; **Location:** stateProvince: Province of Arezzo [AR]; county: Italy; municipality: near Passo la Calla - Stia; **Identification:** identifiedBy: S.N. Wijesinghe; **Event:** year: 2018; month: December; day: 3; habitat: on a dead and aerial stem of *Urtica dioica* (Rosales, Urticaceae); fieldNotes: Terrestrial; **Record Level:** institutionID: MFLU 18-2685; institutionCode: Mae Fah Luang University Herbarium (MFLU); ownerInstitutionCode: IT 4110
- **Type status:** Other material. **Record Level:** type: living culture; collectionID: MFLUCC 20-0155; collectionCode: Mae Fah Luang Culture Collection (MFLUCC)

#### Description

**Figure 5.**
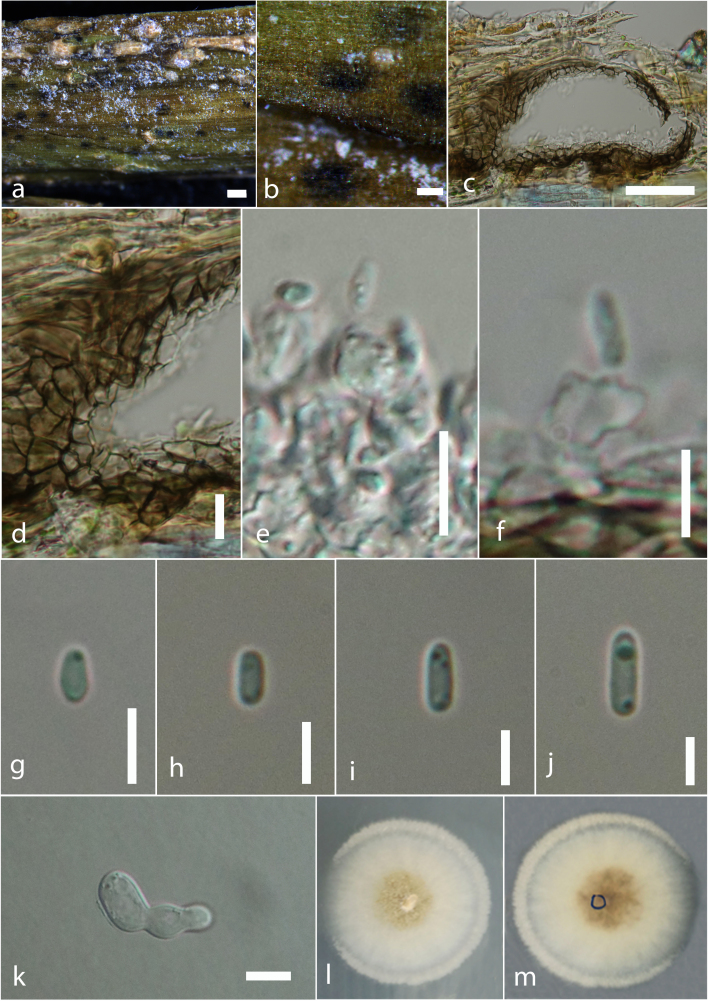
*Phomatodes nebulosa* (MFLU 18-2685). **a-b.** Conidiomata on a dead stem of *Urtica dioica* Rosales, Urticaceae). **c.** Longitudinal section of a conidioma. **d.** Conidiomatal wall. **e-f.** Development stages of conidiogenesis. **g-j.** Conidiospores. **k.** Germinating conidium. **l-m.** Colonies on PDA (**l** upper, **m** lower). Scale bars: a = 100 µm, c = 50 µm, b, k = 20 µm, d-e = 10 µm, f-j = 5 µm.

Saprobic on dead aboveground stem of *Urtica dioica* L. (Rosales, Urticaceae). **Asexual morph**: Coelomycetous. *Conidiomata* (Fig. 5a-c) immersed, raised as black spots on the host surface, pycnidial, 60–70 × 140–170 µm (x¯ = 66.5 × 155 µm, n = 10), solitary, scattered, unilocular, globose or subglobose to irregular. *Pycnidial wall* (Fig. 5d) pseudoparenchymatous, 3–5-layered, 15–30 µm (x¯ = 25 µm, n = 10) wide, thick walled, the outermost layer comprising dark brown cells of *textura angularis*, the inner layer comprising pale brown to hyaline cells of *textura angularis*. *Conidiophores* reduced to conidiogenous cells. *Conidiogenous cells* (Fig. 5e-f) 4–5 × 2–4 µm (x¯ = 4.5 × 3.6 µm, n = 5), enteroblastic, phialidic, ampulliform or short cylindrical, determinate, smooth, hyaline. *Conidia* (Fig. 5g-j) 4–7 × 1–2 µm (x¯ = 5.3 × 1.6 µm, n = 30) ellipsoidal to cylindrical, aseptate, guttulate, smooth-walled, hyaline. **Sexual morph**: Undetermined.

Culture characteristics: Conidia germinating on PDA within 24 h, from single-spore isolation. Colonies (Fig. 5l-m) on PDA reaching 5–10 mm diam. after 10 days at 18°C, circular, entire edge, flat, dense, white in both upper and lower sides.

GenBank accession numbers (ex-MFLUCC 20-0155): ITS = MT880293, LSU = MT880295, TUB2 = MT901291

#### Notes

*Phomatodes* was introduced by Chen et al. (2015) to accommodate *Phoma*-like taxa in Didymellaceae. The type species, *Phomatodes aubrietiae*, is characterised by globose to subglobose pycnidia, ostiolate conidiomata, solitary or confluent, with a 3–5-layered, pigmented pseudoparenchymatous pycnidial wall, phialidic, hyaline, smooth, ampulliform to doliiform conidiogenous cells and cylindrical to allantoid, hyaline, thin-walled, smooth, aseptate, polar guttulate conidia (Chen et al. 2015). The morphology of our material (Fig. 5) agrees with that of the holotype (CBS 100191), with globose to subglobose conidiomata; phialidic, ampulliform conidiogenous cells; and hyaline, aseptate and polar guttulate conidia (5–7 × 1.5–2.5 µm).

From the comparison of ITS, LSU and TUB2 sequences between *P. nebulosa* (CBS 100191-type) and *P. nebulosa* (MFLUCC 20-0155), both strains were identical. In our multi-locus phylogenetic analyses, the new isolate (MFLUCC 20-0155) and the ex-type strains of *P. nebulosa* (CBS 117.93, CBS 740.96, CBS 100191, MFLU 18-0177) clustered together with high support (99 ML/1.00 PP) (Fig. 2).

Early records of *Phomatodes nebulosa* were reported on *Armoracia rusticana* (Brassicales, Brassicaceae) and *Mercurialis perennis* (Malpighiales, Euphorbiaceae) from the Netherlands, *Thlaspi arvense* (Brassicales, Brassicaceae) from Poland (Chen et al. 2015, Farr and Rossman 2020) and *Datisca cannabina* (Cucurbitales, Datiscaceae) from Uzbekistan (Gafforov 2017, Farr and Rossman 2020). Our new strain of *P. nebulosa* from *U. dioica* was collected from the Province of Arezzo in Italy at higher altitude (296 m a.s.l.), compared to the previous Italian record on the same host, but from the Province of Forlì-Cesena (34 m a.s.l.) (Hyde et al. 2020a). Considering the results of our integrative taxonomic approach, we report this strain as a new record of *P. nebulosa*, the first for the Province of Arezzo and the second for Italy, widening its geographic distribution in the country.

## Discussion

**Figure 6.**
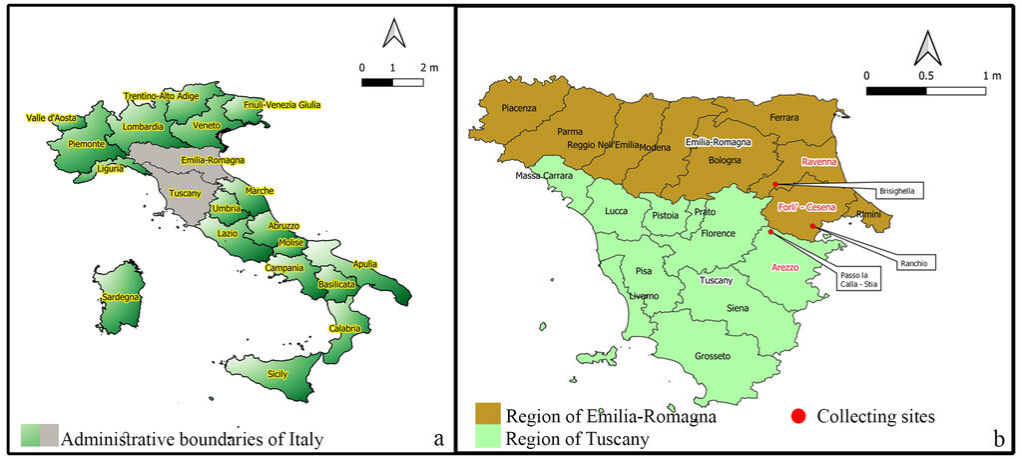
Geographical distribution of newly-isolated species in Italy. **a.** Administrative boundaries of Italy and collection regions (grey). **b.** More detailed map of the collection sites within the Provinces of Arezzo (Tuscany) and Ravenna and Forlì-Cesena (Emilia Romagna).

The pleosporalean fungal collections in this study originated from terrestrial habitats in the Provinces of Arezzo (Tuscany region), Forlì-Cesena and Ravenna (Emilia-Romagna region) in Italy (Fig. 6). The fungal isolates were associated with hosts in Apiaceae, Asteraceae and Urticaceae, which are economically and ecologically valuable plants (Simpson 2010, Bennett 2011). The expansion of ecological and mycogeographical knowledge, other than the taxonomic knowledge, are prerequisites to understand fungal biology, diversity and conservation. Our new species (*Italica heraclei*) and the new record (*Pseudoophiobolus mathieui*) of Phaeosphaeriaceae, reported in this study, led to an expansion of knowledge about the family Phaeosphaeriaceae. *Pseudoophiobolus mathieui* strain was found on a new host, *Artemisia* sp. (Asterales, Asteraceae), enlarging the host distribution of this species in Italy. The record of *Phomatodes nebulosa* (Didymellaceae) for the Province of Forlì-Cesena represents the second record for Italy, widening the geographical range for this species.

At times, members of these fungal families are able to have pathogenic relationships with different host plants in different environments (Hongsanan et al. 2020). Therefore, the accurate reporting of host-fungal records with their geographical locations is highly recommended to gain a better understanding of emerging plant pathogens (Dugan et al. 2009). In this study, we highlighted the expansion of the taxonomic framework and host-fungal relationships of those studied taxa in different Italian geographic regions. Additionally, we combined morphological data and multi-locus phylogenetic analyses to verify their identities and assess their taxonomic placement amongst other pleosporalean taxa.

## Supplementary Material

XML Treatment for Italica heraclei

XML Treatment for Pseudoophiobolus mathieui

XML Treatment for Phomatodes nebulosa

## Figures and Tables

**Table 1.** Gene regions, primers and PCR thermal cycle programmes used in this study, with corresponding reference(s).

**Genes/loci**	**PCR primers** **(forward/reverse)**	**PCR conditions**	**Reference(s)**
ITS and LSU	ITS5/ITS4 and LR0R/LR5	94°C; 2 min (95°C; 30 s, 55°C; 50 s, 72°C; 90 s) × 35 thermal cycles, 72°C; 10 min.	White et al. (1990), Vilgalys and Hester (1990), Hopple (1994), Rehner and Samuels (1994)
SSU	NS1/NS4	95°C; 3 min (95°C; 30 s, 55°C; 50 s, 72°C; 30 s) × 35 thermal cycles, 72°C; 10 min.	White et al. (1990)
TEF	EF1-983F/EF1-2218R	94°C; 2 min (95°C; 30 s, 58°C; 50 s, 72°C; 1 min), × 35 thermal cycles, 72°C; 10 min.	Rehner (2001)
TUB2	Bt2a/Bt2b	94°C; 2 min (94°C; 1 min, 58°C; 1 min, 72°C; 90 s), × 35 thermal cycles, 72°C; 10 min.	Glass and Donaldson (1995)
